# Observations on the Medicinal Uses of the Oleum Fecoris Aselli, or Cod Liver Oil, in the Chronic Rheumatism, and Other Painful Disorders

**Published:** 1782

**Authors:** 


### SECTION II.

### Essays and Observations.

I. *Obſervations on the medicinal uſes of the Oleum Jecoris Aſelli, or Cod Liver Oil, in the Chronic Rheumatiſm, and other painful diſorders. By* Thomas Percival, M.D. F.R.S. *and* S.A. *Member*

*Member of the Royal Society of Physicians at Paris, and of the Medical Societies of London and Edinburgh*, &c. *Read* Oct. 7, 1782.

THE multiplicity of articles, which constitute the materia medica, has been a subject of complaint with some physicians: and, though it is an evil of no great magnitude, it certainly requires correction and reformation. For it must be acknowledged, that many of these articles are known only by their names; and that others are so seldom prescribed, as scarcely to merit the places which they retain in the officinal lists. The progressive accumulation, however, of inactive remedies, is not to be deemed an argument against, but an incitement to the introduction of new ones, which are more efficacious. And, I trust, it will be doing some service to the healing art, to communicate to the public, a brief account of the *oleum jecoris Aselli*, or cod liver oil; the salutary properties of which, I believe, have been little experienced beyond the vicinage of Manchester.

This medicine is dispensed so largely in the hospital here, that near a hogshead of it is annually consumed. It is given in obstinate chronic rheumatisms, sciaticas of long standing, and in in thofe cafes of premature decrepitude, which originate from immoderate labour, repeated ftrains and bruifes, or expofure to continual dampnefs and cold; by which the mufcles and tendons become too rigid, and the flexibility of the joints is impaired, fo as to crackle for want of a due fecretion of fynovia. While I was one of the phyficians to this charity, I had the fulleft evidence of the fuccefsful exhibition of cod liver oil, in various maladies of the clafs above defcribed, which had refifted other powerful modes of treatment. And I frequently compared its operation with that of gum guaiacum, by prefcribing each, at the fame time, to different patients in fimilar circumftances. Thefe trials almoft always terminated in favour of the oil; and the patients, who took guaiacum, by conferring with their fellow-fufferers, were fometimes fo fenfible of making a flower progrefs towards recovery, as to requeft a change of one remedy for the other.

At firft it occafions, for the moft part, an increafe of pain; but this effect fhortly ceafes, and a gradual abatement of the fymptoms fucceeds. The pulfe, in irritable habits, is fometimes accelerated by it, and a glow of warmth has been felt through the whole body, after each dofe of the the medicine. It is neither uniformly laxative, nor binding; but often promotes a gentle degree of perſpiration. However, it proves ſucceſsful, even when it produces no ſenſible operation, as generally happens in perſons habituated to its uſe. In a few weeks, the appetite is impaired by it, the tongue grows foul, and an emetic is required. The doſe of it varies from one table ſpoonful to three, and it may be adminiſtered twice, thrice, or four times daily. In many caſes, it is found ſerviceable to rub the parts affected with the oil, during the courſe of its internal exhibition. But this practice is only to be followed, when no great ſoreneſs ſubſiſts. Indeed, either fever or inflammation forbids the uſe of it entirely.

Cod liver oil is chiefly brought from Newfoundland. It forms a conſiderable article of merchandize; and comes in barrels from 400 to 520 lbs. in weight. The method of obtaining it is, by heaping together the livers of the fiſh, from which, by a gentle putrefaction, the oil flows very plentifully. A ſimilar oil is procured from the livers of the fiſh called ling, and alſo from a ſmall ſpecies of cod found on the coaſt of Buchan in the north of Scotland. The taſte is nauſeous, and leaves upon the palate a ſavour, like that of tainted fiſh. On this account, it is not much preſcribed here, in private practice, amongſt the higher orders of people: but the hoſpital patients make no complaints of it; and ſuch is their confidence in its efficacy, that they often ſolicit, as I before obſerved, to take it; and generally perſevere, with ſteadineſs, in the uſe of it. Indeed we know, that oil, of the ſame kind, forms no inconſiderable part of the food of the Laplanders, and other northern nations. For habit ſoon reconciles to the taſte the moſt diſguſting viands. The cod liver oil may, however, be rendered much leſs offenſive by the following mode of adminiſtering it. ℞. *Ol. Jecoris Aſelli Unciam unam, Aq. Menth. Pip. Simp. Semunciam, Lixiv. Sapon. gutt. XL. Miſce; fiat Hauſtus.* By this combination a liquid ſoap, not very unpleaſant, is produced, which may be readily decompoſed by the addition of a tea-ſpoonful of the juice of lemons. And as the oil is probably moſt efficacious in its original form, it may be adviſable to drink a cup of ſome acidulous liquor immediately after the medicine has been ſwallowed. This will at once cleanſe the mouth and gullet, neutraliſe the alkaline ſalt, and ſeparate the oil in the ſtomach. Dr. Ruſſel, in his natural hiſtory of Aleppo, has obſerved, that " in

" certain

" certain ſeaſons, when oil is plentifully taken, " the people there become diſpoſed to fevers " and infarctions of the lungs, which ſymptoms " wear off by retrenching this indulgence." I have never ſeen or heard of any ſuch effects from the long continued uſe of the oleum jecoris Aſelli. Perhaps this diverſity may partly depend on the different qualities of vegetable and fiſh oil; the former having a tendency to obſtruct, the latter to promote inſenſible perpiration. But, I apprehend, it is chiefly to be aſcribed to the influence of climate. The intenſe heats of Turkey relax the animal fibres, and oil adds to this relaxation. But, under a northern ſky, the fibres are too much diſpoſed to rigidity, and when this actually ſubſiſts, as a malady, the emollient powers of oil are ſo far from being injurious, that they are highly ſalutary.

As my chief deſign in announcing the virtues of the *oleum Aſelli*, is to recommend its uſe in hoſpitals, diſpenſaries, and pariſh work-houſes, I ſhall ſubjoin the following letter, written, at my deſire, by Mr. Darbey, houſe-ſurgeon and apothecary to our Infirmary. The teſtimony which it bears in favour of this medicine, is the more forcible, as it is founded not on the experience of one, but of all the phyſicians to the charity; whose patients he daily attends and examines. And it is but justice to him to add, that he is a man of judgment, observation, and integrity.

" To Dr. Percival.

" Dear Sir,

" IN compliance with your request, I send " you the following remarks on some rheumatic " cases:

" For several years after I came to the In- " firmary, I observed that many poor patients, " who were received into the Infirmary for the " chronic rheumatism, after several weeks trial " of a variety of remedies, were discharged with " little or no relief. The volatile tincture of " guaiacum, in large doses, seemed to bid the " fairest to effect a cure; but the success did " not answer expectation. About ten years " since, an accidental circumstance discovered " to us a remedy, which has been used with " the greatest success, for the above complaint, " but is very little known in any county except " Lancashire: it is the Cod, or Ling liver oil.

" A woman, who laboured under the most " excruciating rheumatism, and was an out- " patient of this Infirmary, being advised to rub

" her

"her joints with the oil, was induced to take it, "at the ſame time, internally. A few weeks "reſtored her to the uſe of her limbs, and ſhe "was cured. However, little attention was "paid to this caſe, as it was ſuppoſed that the "alteration of the weather, and the medicines "ſhe had before taken, had cauſed the cure. "About a twelvemonth afterwards, her com- "plaints returned with double violence, and the "ſame remedy reſtored her to health again.

"Encouraged by this ſecond recovery, Dr. "Kay, one of the phyſicians to the Infirmary, "preſcribed it for other patients in ſimilar caſes, "and it anſwered his moſt ſanguine expectations. "Since then, it has been uſed by the other "phyſicians with the greateſt ſucceſs. One "diſadvantage attending the uſe of the fiſh oil "is, its nauſeous ſmell and taſte; this is ſo bad, "that, I am afraid, many delicate ſtomachs "cannot take it: yet I have obſerved that the "poor, after taking a few doſes, readily drink "it without any appearance of nauſea, and that "not more than one ſtomach in twenty rejects it. "This medicine has very different effects on "different conſtitutions: on ſome it operates as "a purgative; others are coſtive with it; and "ſometimes it occaſions a gentle ſweat. When

"the

" the latter has been the caſe, the cure has been " more certain and expeditious; though it muſt " be obſerved, that the uſe of it, for the firſt " week or fortnight, ſeems to occaſion a general " increaſe of pain.

" Men and women, advanced in years, whoſe " fibres may be ſuppoſed to have acquired a " degree of rigidity, find ſurpriſing effects from " it. Some, who have been cripples for many " years, and not able to move from their ſeats, " have, after a few weeks uſe of it, been able " to go with the aſſiſtance of a ſtick; and, by a " longer continuance, have enjoyed the pleaſing " ſatisfaction of being reſtored to the natural " uſe of their limbs, which, for a long time " before, had been a burthen to them.

" Two caſes occurred lately, in which the oil " had an extraordinary effect, even on young " perſons, whoſe ages did not exceed ten years. " Guaiacum, calomel, bliſters, &c. were tried " on both theſe patients, but with ſo little benefit " that opiates were given merely to procure tem- " porary eaſe. Their lower limbs ſeemed to be " a burthen to them, and they had ſuch an ap- " pearance of diſtortion that no hopes of relief " could be well entertained. In compliance " with the particular requeſt of their parents,

" the

"the cod oil was given; the one obtained a

"perfect cure, the other nearly so; the latter

"having a little distortion in his back, is pre-

"vented the free use of his legs. So general

"has been the use of the oil with us, that we

"dispense fifty or sixty gallons annually; and

"the good effects of it are so well known

"amongst the poorer sort, that it is particularly

"requested by them for almost every lameness.

"Except bark, opium, and mercury, I believe

"no one medicine in the materia medica is likely

"to be of greater service. Besides, there is one

"other circumstance which recommends this

"medicine, which is, that it is a cheap one:

"and I could wish for a more general use of it,

"in order to prove that the above account of

"its good effects is no exaggeration.

"I am, Sir,

"Your obliged humble servant,

"ROBERT DARBEY.

*Manchester Infirmary,*

*Feb.* 12, 1782.

